# Synchronization-based fusion of EEG and eye blink signals for enhanced decoding accuracy

**DOI:** 10.1038/s41598-024-78542-9

**Published:** 2024-11-06

**Authors:** Emad Alyan, Stefan Arnau, Julian Elias Reiser, Edmund Wascher

**Affiliations:** Department of Ergonomics, Leibniz Research Centre for Working Environment and Human Factors, 44139 Dortmund, Germany

**Keywords:** Cognitive neuroscience, Computational neuroscience, Human behaviour

## Abstract

Decoding locomotor tasks is crucial in cognitive neuroscience for understanding brain responses to physical tasks. Traditional methods like EEG offer brain activity insights but may require additional modalities for enhanced interpretative precision and depth. The integration of EEG with ocular metrics, particularly eye blinks, presents a promising avenue for understanding cognitive processes by combining neural and ocular behaviors. However, synchronizing EEG and eye blink activities poses a significant challenge due to their frequently inconsistent alignment. Our study with 35 participants performing various locomotor tasks such as standing, walking, and transversing obstacles introduced a novel methodology, pcEEG+, which fuses EEG principal components (pcEEG) with aligned eye blink data (syncBlink). The results demonstrated that pcEEG+ significantly improved decoding accuracy in locomotor tasks, reaching 78% in some conditions, and surpassed standalone pcEEG and syncBlink methods by 7.6% and 22.7%, respectively. The temporal generalization matrix confirmed the consistency of pcEEG+ across tasks and times. The results were replicated using two driving simulator datasets, thereby confirming the validity of our method. This study demonstrates the efficacy of the pcEEG+ method in decoding locomotor tasks, underscoring the importance of temporal synchronization for accuracy and offering a deeper insight into brain activity during complex movements.

## Introduction

The human brain is a sophisticated organ that continually processes an immense amount of information from both its internal and external environments. This dynamic processing is propelled by neuronal activity, which can be decoded through multivariate pattern analysis (MVPA) to uncover the stimuli or mental states triggering such activities^1,2^. A deep connection exists between movement and complex cognitive processes, highlighting a balanced relationship essential to our engagement with the environment. Indeed, performing cognitive tasks during locomotion is a common aspect of our daily existence, showcasing the brain’s multitasking ability. However, there remains a significant research gap in understanding how brain activity, especially during dynamic real-world tasks, can be decoded from multiple physiological signals simultaneously, such as brain activity and eye blinks. Current literature has largely focused on decoding brain activity using single modality like electroencephalography (EEG) data. The challenge of “decoding” cognitive actions during movement remains a continuing scientific question, requiring further exploration and clarification^3^.

Previous studies have primarily focused on MVPA using EEG to classify brain activity in response to various stimuli^4^. While such approaches have improved the decoding of cognitive states from EEG signals, they often neglect the contribution of ocular events, particularly eye blinks, which are also reflective of neural processes. Additionally, traditional MVPA approaches, especially those that rely on sliding window models, face limitations in decoding performance, especially when dealing with many-class datasets, as they often fail to capture the full spatiotemporal features of the brain signal^5^. There remain notable limitations regarding the interpretability and synchronization of multiple physiological signals during dynamic tasks. This suggests the need for a more integrated approach that combines multiple physiological signals to decode complex brain states more accurately. The fusion of EEG with other modalities has been explored in some cases, but the focus has often been on classification^6–9^ rather than understanding the neural activity patterns underlying both ocular and brain activity. This study aims to address this by integrating EEG data with involuntary ocular events, such as blinks, to better understand brain-ocular dynamics during multitasking.

Recent studies show that spontaneous eye blinks are more than a mere physiological necessity; they are a subtle yet significant indicator of cognitive processing and attentional shifts^10^, reflecting the underlying neural mechanisms and states. Research has revealed a significant connection between blink rate and the dopaminergic system, where increased blink rates are associated with higher dopaminergic activity, as observed in schizophrenic patients, and vice versa in Parkinson’s patients^11,12^. Furthermore, lower blink rates have been associated with heightened attention and cognitive flexibility, highlighting a link between blinking patterns and the dopaminergic system^13,14^. Joint analysis of eye blinks and brain activity during tasks like visual search reveals a strong link between blink patterns and cognitive processing^15^. Blinks often occur during natural pauses in information flow^16,17^, which could suggest that they signal the end of a cognitive phase and facilitate shifts from external attention to internal processes like memory retrieval^17^. These findings highlight the importance of combining eye blink signals with EEG to better understand and distinguish between neural mechanisms of different real-world scenarios.

Most existing studies have used external sensors such as eye trackers or electrooculography (EOG) to capture eye blink data^6–9^, which requires synchronization between external sensors and EEG signals. Raw EEG data is often tainted by noise and artifacts, including interference from eye blinks, which heightens the challenge of decoding brain activity. However, recent studies have begun to see involuntary eye blinks, extracted from EEG, in a new light—suggesting they may play roles in cognitive and visual processing, potentially serving as event markers to distinguish between various cognitive states^16,18–20^. For instance, Wascher et al.^21^ highlighted the cognitive significance of blinks, suggesting that they are not merely physiological artifacts but carry information about cognitive processes.

This emerging understanding of involuntary eye blinks as potentially informative, rather than merely disruptive, marks a significant paradigm shift in EEG data interpretation. It accentuates the necessity for advanced analytical techniques in EEG research. To effectively navigate these complexities and decode brain activity with precision, it’s imperative to utilize methodologies capable of isolating the most pertinent components within the EEG data. Among such methodologies, Principal Component Analysis (PCA)^22^ plays a crucial role. Given the significant redundancy often present in EEG data, the application of PCA is essential to extract the most informative components, thereby eliminating extraneous information. PCA, widely accepted for complexity reduction, particularly in high-dimensional datasets like EEG recordings, converts the original dataset into a series of linearly uncorrelated variables, known as principal components (PCs). The first few PCs extracted using PCA capture most of the variance in the data, making them optimal features for classification tasks. Unlike traditional time or frequency domain methods that require manual selection of features, PCA allows for automated, data-driven feature extraction by identifying the most informative components. This not only improves classification performance but also reduces noise and offers an accurate representation of neural data^23^. Recent studies demonstrate that PCA has been effective in improving classification accuracy in EEG-based tasks, such as emotion classification^24^ and motor imagery^25^.

Recently, there has been an increasing trend in the integration of data from multiple sensors or sources across various scientific disciplines, enhancing the comprehensive understanding of complex phenomena. This trend is particularly prominent in neuroscience, where the combination of EEG data with other modalities like pupillometry and eye tracking shows promise in classifying mental states and assessing cognitive workload^7,8^. For example, Borys et al.^7^ investigated the fusion of EEG and pupillometry features for mental workload assessment, employing various classification algorithms. Interestingly, they found that eye-tracking features alone yielded higher accuracy than the combined modalities. Brouwer et al.^8^ also explored the complementarity of EEG and pupillometry, concluding that the integration of both modalities led to improved accuracy in cognitive workload evaluation. Delving into emotion classification using EEG and eye tracking, Zheng et al.^26^ compared feature-level fusion against multimodal deep learning techniques, with the latter showing superior performance. Building further on these findings, Shahbakhti et al.^27^ demonstrated that blink-related features, when combined with EEG data and analyzed through an SVM using the Fp1 and Fp2 channels, significantly improved driver fatigue detection accuracy. In a subsequent study, the authors introduced a driver fatigue detection technique using EEG and eye blinks and tested its robustness across two databases^28^. However, these fusion studies focus primarily on classification tasks, which tend to categorize data into discrete labels, rather than decoding continuous neural-ocular activity patterns related to tasks. This highlights the necessity for decoding approaches that focus on understanding the interplay between brain and ocular systems beyond simple classification.

Despite the growing interest in EEG integration with other modalities, significant gaps remain. A primary issue is the inconsistent synchronization of EEG with ocular events, particularly blinks. Alyan et al.^29^ revealed that EEG data associated with blinks yields higher classification accuracy compared to random EEG epochs, which highlights the importance of this synchronization. However, the peak timing of EEG decoding after the peak of the blink peak may vary depending on the task, while blink-related metrics show higher accuracy during eye closure and reopening, with a slight advantage during closure^20^. This temporal discrepancy suggests an optimal post-blink time window for capturing relevant EEG data, emphasizing the need for precise temporal alignment. Another promising avenue is determining the temporal lag between the peak of EEG activity—measured as the global field power (GFP) peak, which reflects the time point associated with the largest evoked response following a blink—and the peak of the blink. Accurate determination of this lag could significantly improve EEG-blink alignment and decoding accuracy.

Considering the research gaps and opportunities, our study seeks to leverage the synergy between EEG data and synchronized eye blink information during locomotion tasks, including standing, walking, and navigating obstacles. We propose a new methodology that fuses these two modalities, optimizing the temporal alignment between eye blinks and peak EEG activities. The objective is to explore whether the fusion-based synchronization of EEG and eye blink data enhances decoding accuracy compared to single modalities, particularly during locomotion tasks. Our approach involves analyzing EEG signals using principal components that are then concatenated with eye blink data. In addition, we will test and compare this method against canonical correlation analysis (CCA), which identifies the maximally correlated variables between EEG and eye blink data^30^. This approach is predicated on the hypothesis that a more nuanced temporal synchronization of EEG signals with eye blink dynamics will substantially enhance decoding accuracy, particularly in dynamic tasks involving both static and movement-based activities. Recent studies have demonstrated the efficacy of CCA in fusing EEG with modalities like fNIRS and fMRI to enhance classification performance^31,32^. The use of CCA ensures that the extracted features capture the shared variance; thus, enhancing the fusion process and ultimately improving the overall decoding performance.

Our method will be validated using two distinct driving datasets, each containing three levels of difficulty: one focused on proactive driving, the other on reactive driving. These driving tasks were chosen because they demand continuous motor and cognitive integration, similar to locomotor tasks, but with different complexity and task variability. By incorporating these tasks, we aimed to test whether our model could generalize across different forms of movement and task types, thus addressing the research question of how well multimodal fusion models perform beyond locomotor contexts. We expect this multimodal integration to provide a further understanding of cognitive states during physical tasks, surpassing single-modality analyses. We hypothesize that this approach will significantly improve decoding accuracy across conditions and offer deeper insights into the relationship between cognitive states and physical activities.

## Materials and methods

### Participants

We performed an analysis of the data obtained from two experiments published in^33,34^. A total of 39 eligible participants, who were right-handed, free of any past or current neurological or psychiatric disorders, and possessed either normal vision or corrected-to-normal vision, were incorporated in the preprocessing. Four participants were eliminated due to insufficient data quality and the challenge of precisely quantifying blinking behavior, which led to a decrease in the total number of recorded epochs. The remaining participants, consisting of 18 females and 17 males, had an average age of 23.51 with a standard deviation (σ) of 2.95. Prior to their participation, all individuals provided written consent and were compensated at a rate of EUR 10 per hour for their involvement. Both research projects obtained approval from the local ethics committee of the Leibniz Research Centre for Working Environment and Human Factors (IRB approval number: 121-2018) and were conducted following the Declaration of Helsinki.

### Procedure and stimuli

The two experiments employed well-established auditory paradigms and delivered stimuli through passive noise-canceling in-ear headphones (Bose QuietComfort 20). In the first experiment^34^, participants undertook an auditory oddball task, distinguishing rare target sounds among frequent standard ones. This task entailed identifying a deviant high-pitched (900 Hz) sinusoid tone amidst regular low-pitched (600 Hz) ones. Participants pressed a button for deviant tones and abstained from responding to standard ones. The second experiment^33^ employed an auditory task-switching paradigm. Participants received a cue tone, either low (600 Hz) or high (900 Hz), followed by a spoken German number ranging from one to four or six to nine. Based on the cue, they indicated whether the number was below or above five or if it was odd or even. This involved a 5-minute repeat task or a 10-minute switch task with varying locomotor complexities.

**Fig. 1:**
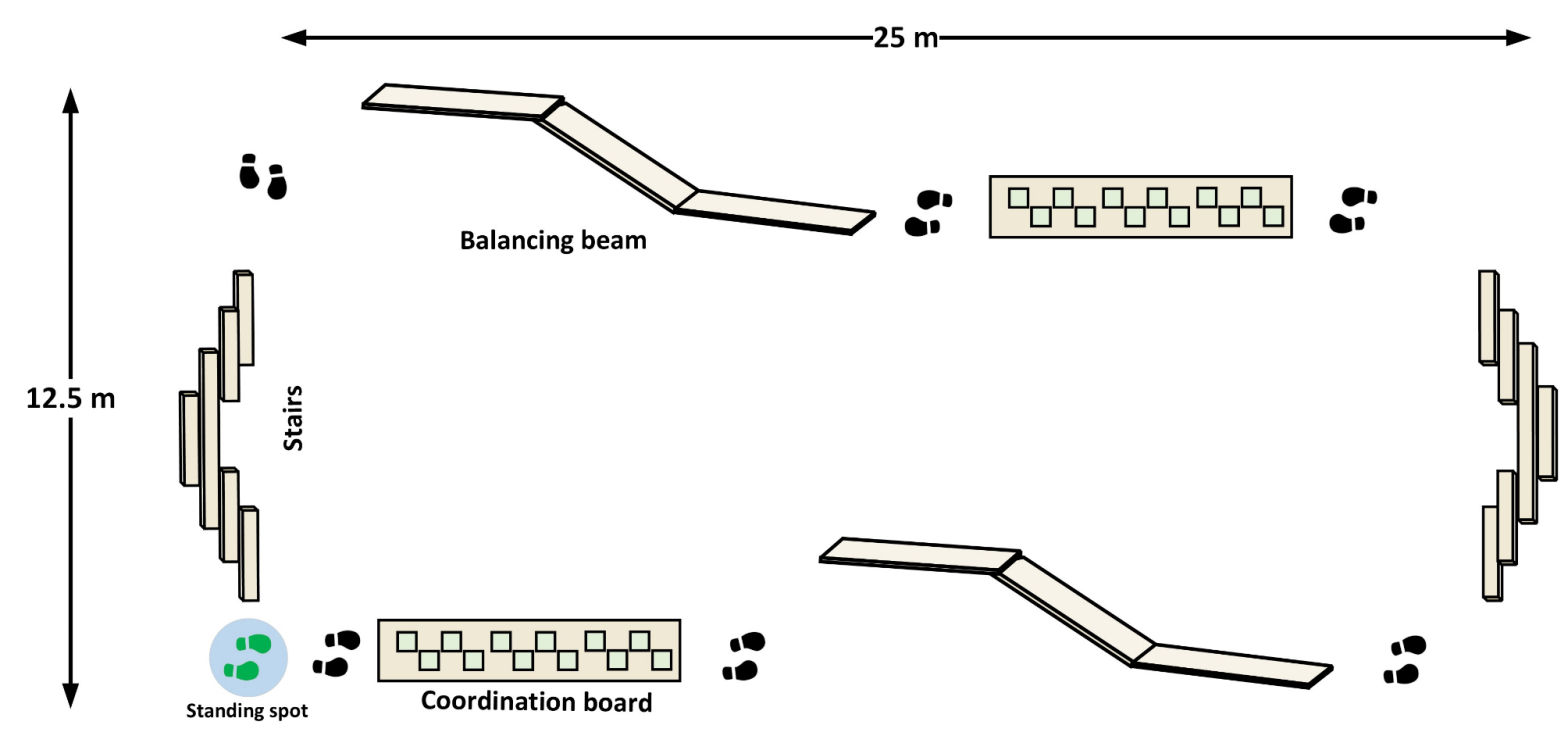
Illustration of the obstacle course layout. The shown course has a continuous perimeter of 75 m and includes pairs of staircases, balancing beams, and coordination boards with foot holes. Participants must ascend and descend the stairs, maintain balance on the beams without touching the ground, and navigate the coordination boards by placing their feet in the designated cut-out holes. The grey circle marked with green footsteps denotes the designated standing area.

Both experiments incorporated three distinct locomotor complexity conditions executed in an outdoor environment. Participants either stood still at a specified spot, walked around the obstacle course at a comfortable pace, or navigated through obstacle elements within the course (refer to Fig. 1). The obstacle course featured staircases, balancing beams, and balancing boards. The duration for each locomotor condition mirrored the auditory tasks’ presentation time. Overall, participants dedicated a total of 90 min to the experiment, with the time equally distributed among the locomotor tasks. Both clockwise and anti-clockwise directions were explored during walking and obstacle navigation. The sequence of walking tasks was quasi-randomized using a Latin square design, ensuring they were executed in both halves of the experiment.

### Data acquisition

EEG data were collected using a 10–20-system montage with 30 electrodes. FCz was the online reference, and AFz was the ground electrode. After placing a cap with electrode holders on the participant, electrodes were filled with conductive gel and adjusted until the impedances fell below 10 kΩ. Care was taken to ensure cables did not cross or move to avoid electric motion artifacts. Data was routed to a mobile LiveAmp EEG amplifier (Brain Products GmbH, Gilching, Germany) placed in the cap’s pocket, recording at 500 Hz with a 24-bit depth onto a micro-SD card. Concurrently, this data was monitored on a laptop through the amplifier’s Bluetooth connection using Brainvision Recorder software. Post-experiment, data was transferred from the SD card to the laptop with the LiveAmp File Converter.

### Replication datasets

To replicate our findings, we utilized two datasets from distinct driving experiments, namely reactive and proactive, conducted with a stationary driving simulator. Each experiment included 29 participants (15 females and 14 males), selected after data quality and blinking behavior considerations. The average age was 42.6 years (σ = 20.2) for the reactive group and 41.1 years (σ = 20.0) for the proactive group. All participants were regular car users and underwent health screenings prior to consenting to the study, which conformed to ethical standards. In the reactive driving experiment, participants navigated a two-lane road under simulated crosswinds, generated by varying road slopes and sinusoidal lateral forces. The proactive driving required maintaining precise control on a single-lane road with varying curve radii. Both scenarios were set in a grassy, distraction-free environment at a consistent speed of 31 mph. The study manipulated task difficulty through three levels of crosswind amplitude and curve radii, presented in random order over 10 sets, including an initial training session, for a total of 54 uninterrupted minutes. Further details are available in references^20,35,36^. Both studies were approved by the local ethics committee of the Leibniz Research Centre for Working Environment and Human Factors and conducted in compliance with the Declaration of Helsinki.

### The proposed method

**Fig. 2:**
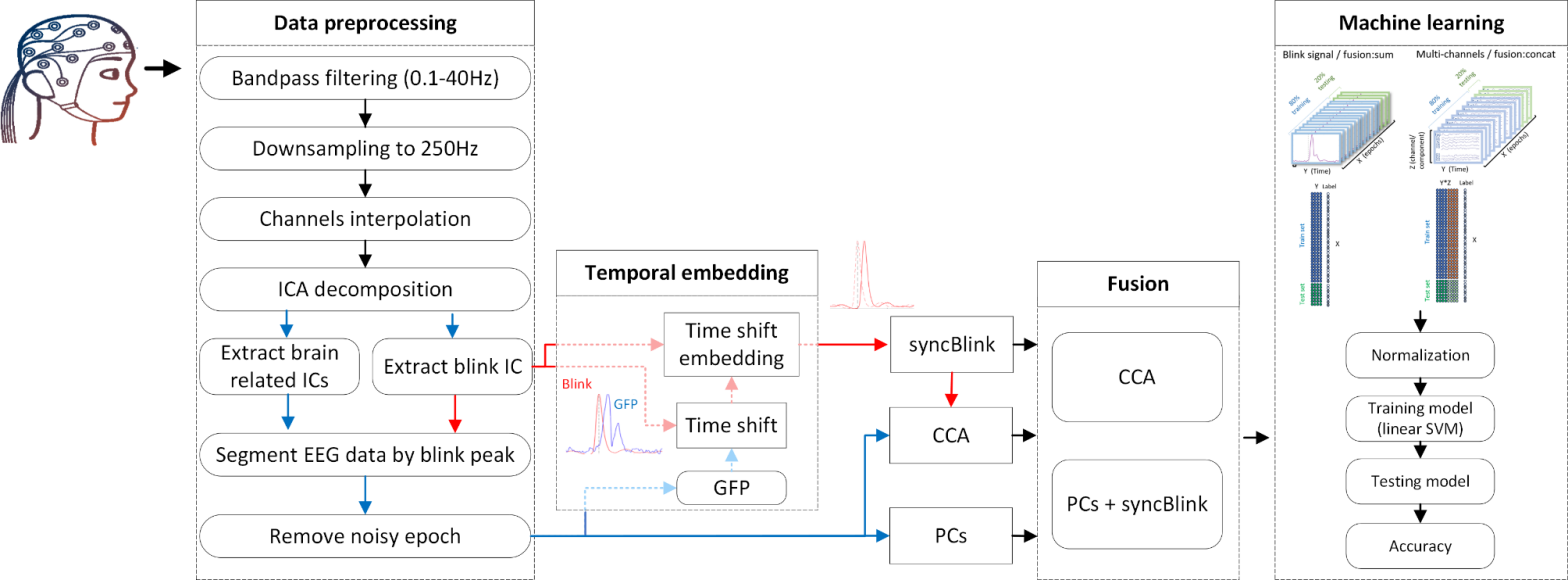
Illustration of the proposed framework for integrating EEG and eye blink data, which includes: (1) EEG data preprocessing and eye-blink extraction, (2) Fusion of EEG data with the aligned blink signal, (3) Classification and multivariate pattern analysis.

The proposed method is depicted in Fig. 2. Initially, the raw EEG signal underwent band-pass filtering to minimize noise and was re-sampled to lessen computational demands. Concurrently, eye-blink extraction was conducted. Subsequently, the eye-blink data was aligned with the GFP-EEG prior to the fusion of synchronized blink signals with EEG signals. Ultimately, a selected subset of features was introduced to classifiers for multivariate pattern analysis, distinguishing between various locomotor complexity conditions. Detailed descriptions of this method are provided in the following subsections.

#### Preprocessing

The data was analyzed using custom scripts from EEGLAB, executed on MATLAB^37^. Initially, to mitigate the effects of environmental and muscular artifacts, the raw EEG data was filtered with finite impulse response (FIR) filters (eegfiltnew) using a high-pass filter at 0.1 Hz and low-pass filters at 40 Hz. The data was then down-sampled to 250 Hz, after which the “clean_artifacts” function was employed to detect and label unwanted channels using default parameters (flatline = 0.5s, burst = 5, line noise = 4, correlation = 0.8, and window = 0.25). After re-referencing the data to a common average, a high-pass zero-phase Hamming window FIR filter with a 1.5 Hz cutoff frequency was applied. This filtering process was followed by the decomposition of the data into statistically independent components (ICs) through the AMICA algorithm^38^. The resulting ICs were then superimposed on the average referenced data, representing the data before the high-pass filtering stage. In the next phase, the IClabel algorithm^39^ was utilized on the decomposed signal to autonomously classify the components, discarding those unrelated to brain activity. Specifically, components that had less than 30% brain association and more than 30% other classification probabilities.

On the other hand, the study aimed to utilize the blink IC as an event marker and to represent continuous blink signals indicative of eye blink activity. Thus, the blink IC was distinguished from other eye ICs, such as the saccade-related IC. This distinction was made by evaluating the Pearson correlation between the left and right anterior channels of eye ICs categorized by the IClabel algorithm. The IC that exhibited a high positive correlation was identified as the blink-eye IC. Subsequently, this selected eye blink IC was input into the BLINKER tool using default settings. Its primary function, BLINKER, is to identify potential blink intervals in the EEG signal, specifically when the signal exceeds the overall mean by more than 1.5 standard deviations (further details can be found in^40^). This tool detects blinks and integrates relevant blink maximum event markers into the EEG.event data structure to segment the blink (continuous blink IC time series) and brain signal. Afterward, epochs ranging from − 500 ms to 1000 ms relative to the eye blink peak were extracted. These epochs were processed using an EEGLAB function named “pop_autorej” which automatically rejects certain epochs. Any epoch surpassing an absolute threshold value of 500 µV or a standard deviation threshold of 5 was discarded. This removal process was iterative, allowing for a maximum of 10% of epochs to be excluded in each iteration.

#### Feature-level fusion

Given the dynamic behavior of signals in temporal space, one critical aspect to consider is the optimal synchronization between the blink signal and the GFP-EEG patterns. We denote the temporal instances of maximum blink amplitude and the maximum GFP amplitude as ${\text{t}}_{{\text{blink}\_{{\max}}}}$ and ${\text{t}}_{{\text{GFP}\_{{\max}}}}$ respectively. These are derived from the average across all epochs. The temporal offset between these two maxima denoted as ${\Delta}{\text{t}},$ can be formally defined as:

1 $${\Delta}\text{t}={\text{t}}_{{\text{GFP}\_\text{max}}}-{\text{t}}_{{\text{blink}}\_{\text{max}}}$$

To achieve synchronization between the blink signal and the concurrent GFP and EEG patterns, the blink signal, BC or BCS, is temporally shifted by ${\Delta}\text{t},$ as given by:

2 $${\text{BC}}_{\text{sync}}\left(\text{t}\right) = {\text{BC}}\left(\text{t}+{\Delta}\text{t}\right)$$

3 $${\text{BCS}}_{\text{sync}}\left(\text{t}\right)={\text{BCS}}\left(\text{t}+{\Delta}\text{t}\right)$$

Here, BC corresponds to the blink component signal, while BCS is a transformed version of the blink component signal in channel space.

**Fig. 3:**
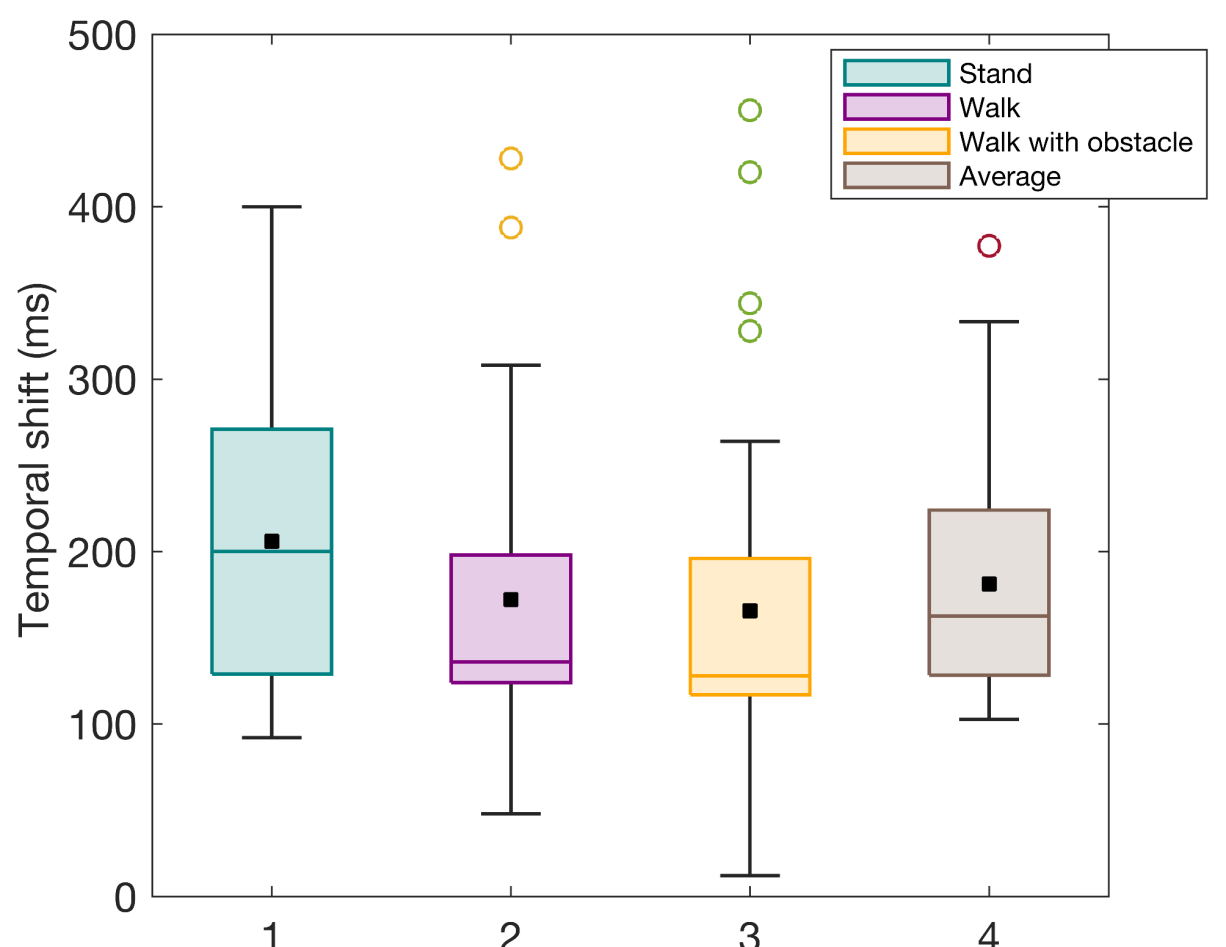
Temporal shifts in GFP peaks following the onset of eye-opening: a comparative analysis across standing, walking, and walking with obstacles conditions.

In this study, the fusion algorithms employ CCA variables that capture both blink and EEG. Also, we integrate, on a per-epoch basis, either the synchronized blink component, ${BC}_{sync}$ or its transformed counterpart ${BCS}_{sync}$, alongside the EEG principal components. Figure 3 illustrates the temporal dynamics of GFP peaks post-eye opening under three locomotion conditions: standing, walking, and transversing obstacles. It presents the central tendency and variability within each condition, using black squares and lines to denote means and medians, respectively. These findings indicate that peak neural activities in response to the blink vary across different locomotor activities. The mean comparisons across conditions reveal systematic neural timing shifts, suggesting the brain’s adaptability to changing locomotor demands. To account for variability in temporal shifts across conditions, we averaged these shifts for each participant, ensuring a standardized alignment process. This method reduces potential classification biases arising from inconsistent temporal alignments.

#### *PCA on EEG data*

PCA is executed on the EEG signal X(t) to extract the principal components that capture 95% of the variance. These components are delineated as:

4 $${{\text{PC}}}_{\text{i}}\left(\text{t}\right)=\left[{{\text{PC}}}_{1}\left(\text{t}\right),{{\text{PC}}}_{2}\left(\text{t}\right),\dots,{{\text{PC}}}_{\text{k}}\left({\text{t}}\right)\right]$$

where k represents the k-th principal component as a vector at time t. The fused signals can be represented through vector concatenation:

5 $${{\text{Fusion}}}_{{\text{concat}}}\left(\text{t}\right)=\left[{\text{PC}}_{\text{i}}\left(\text{t}\right),{\text{BC}}_{{\text{sync}}}\left(\text{t}\right)\right]$$

By extracting principal components, we ensure that the features fed into the classifier capture the most informative variance, enabling more precise neural decoding. These components were then concatenated with eye-blink data. This concatenation creates a unified feature set that integrates information from both brain and ocular signals.

#### *CCA on EEG-Eye blink data*

CCA seeks to uncover canonical variables u and v that maximize the correlation between ${\text{X}}^{{\prime}}={\text{u}}^{\text{T}}{\text{X}}\left({\text{t}}\right)$ and ${\text{B}}^{{\prime}}={{\text{v}}^{\text{T}}{\text{BCS}}}_{{\text{sync}}}\left({\text{t}}\right)$. The optimization target is:

6 $${\text{corr}}\left({\text{X}}^{{\prime}},{\text{B}}^{{\prime}}\right)=\frac{{\text{u}}^{\text{T}}{\text{S}}_{{\text{xb}}}{\text{v}}}{\sqrt{{\text{u}}^{\text{T}}{\text{S}}_{{\text{xx}}}{\text{u}}}\sqrt{{\text{v}}^{\text{T}}{\text{S}}_{{\text{bb}}}{\text{v}}}}$$

where ${\text{S}}_{{\text{xx}}}$ and ${\text{S}}_{{\text{bb}}}$ denote the autocovariance matrices of X’ and B’ respectively, while ${\text{S}}_{{\text{xb}}}$ is the cross-covariance matrix. The denominator acts as a normalization factor, ensuring that CCA remains invariant to coefficient scaling changes. To enforce normalization constraints on u and v, the Lagrange multipliers ${{\uplambda}}_{1}$ and ${{\uplambda}}_{2}$ are introduced. This gives rise to the Lagrangian:

7 $${\text{L}}\left({\text{u}},{\text{v}},{{\uplambda}}_{1},{{\uplambda}}_{2}\right)={\text{u}}^{\text{T}}{\text{S}}_{{\text{xb}}}{\text{v}}-{{\uplambda}}_{1}\left({\text{u}}^{\text{T}}{\text{S}}_{{\text{xx}}}{\text{u}}-1\right)-{{\uplambda}}_{2}\left({\text{v}}^{\text{T}}{\text{S}}_{{\text{bb}}}{\text{v}}-1\right)$$

From this Lagrangian, we derive two eigenvalue problems:

$${\text{For u:}}\; {\text{S}}_{{\text{xx}}}^{-1}{\text{S}}_{{\text{xb}}}{\text{S}}_{{\text{bb}}}^{-1}{\text{S}}_{{\text{xb}}}^{\text{T}}{\text{u}}={{\uplambda}}^{2}{\text{u}}$$

$${\text{For u:}}\; {\text{S}}_{{\text{bb}}}^{-1}{\text{S}}_{{\text{xb}}}^{\text{T}}{\text{S}}_{{\text{xx}}}^{-1}{\text{S}}_{{\text{xb}}}^{\text{T}}{\text{v}}={{\uplambda}}^{2}{\text{v}}$$

Upon solving these, we obtain the canonical variables u and v. With these variables in hand, fusion can be accomplished either through concatenation:

8 $${\text{Fusion}}_{{\text{concat}}}\left(\text{t}\right)=\left[{\text{u}}; {\text{v}}\right]$$

In that, CCA identifies pairs of maximally correlated variables between the two modalities, effectively synchronizing the EEG and blink data, which improves classification performance for locomotor tasks.

#### Classification

Classification using fusion approaches plays a pivotal role in decoding analyses, significantly enhancing the precision of multivariate analyses. These approaches allow for the identification of subtle data patterns that may go unnoticed in single-modality studies. Given this, our research seeks to integrate the dynamics of eye blink signals with EEG signals in the form of principal components. We also incorporate CCA variables that capture both blink and EEG phenomena. A crucial part of our methodology involved aligning the peak of the eye blink with the GFP peak, which is associated with neural activity in response to a blink.

For feature extraction, we employed a moving window of 50 ms integration time, which slid over the segmented signal ranging from − 500 to 1000 ms relative to the onset of the blink. This window covered roughly 13 sampling instances (at our 250 Hz sampling rate), sourced from either standalone EEG, blink signals, or a fusion of both. The assembly of the feature matrix was contingent on the number of electrodes/components of the signal, multiplied by the data points within the 50 ms window and the number of trials. For example, in the case of 5 EEG PCs across 500 trials, the EEG data shape is 65 × 500 (with 65 being the result of 5 PCs × 13 sampling points). When syncBlink data is added, it contributes a shape of 13 × 500 (representing blink signals across the same 13-time points). The fused data, therefore, results in a shape of 78 × 500 (65 EEG time points + 13 syncBlink time points). A linear SVM classifier was then applied to project these features, illuminating the temporal patterns pre- and post-eye blink. This effort was designed to distinguish between conditions over designated periods. To avoid biases and ensure a uniform representation of the classes, we downsampled the majority class to align with the size of the minority class, leading to an average of 467 balanced epochs for each condition.

The classifier’s evaluation was conducted using a five-repetition cross-validation strategy. In this approach, datasets were randomly divided into a training set (comprising 80% of trials) and a testing set (making up the remaining 20%) during each repetition. To ensure consistency in the classification process, feature vectors from the training set, consisting of raw potential values, were normalized using z-score methods. Importantly, the testing data was normalized using parameters (e.g., mean and standard deviation) obtained solely from the training set. The performance of the decoding models is assessed according to the accuracy (Acc), sensitivity (Sen) and specificity (Spe) as follows:

9 $${\text{Acc}}=\left(\frac{{\text{T}}_{\text{P}}+{\text{T}}_{\text{N}}}{{\text{T}}_{\text{N}}+{\text{T}}_{\text{P}}+{\text{F}}_{\text{P}}+{\text{F}}_{\text{N}}}\right)\times100$$

10 $${\text{Sen}}=\left(\frac{{\text{T}}_{\text{P}}}{{\text{F}}_{\text{N}}+{\text{T}}_{\text{P}}}\right)\times100$$

11 $${\text{Spe}}=\left(\frac{{\text{T}}_{\text{N}}}{{\text{F}}_{\text{P}}+{\text{T}}_{\text{N}}}\right)\times100$$

where TN, TP, FP, and FN represent the counts of true negatives, true positives, false positives, and false negatives in a classification model, respectively.

### Statistical analysis

Statistical analyses were conducted utilizing paired t-tests to assess the significance of differences between the proposed methods across various training time points within the temporal generalization matrix. The threshold for statistical significance was set at *p* < 0.05. To reduce the likelihood of Type I errors, the calculated *p*-values underwent a correction for the false discovery rate (FDR)^41^. For a rigorous assessment of statistical significance related to temporal generalization, we employed an extreme pixel-based permutation test. As substantiated by prior empirical studies, this approach provides enhanced sensitivity for the detection of smaller clusters when contrasted with traditional cluster-based permutation tests^42^. Our null hypothesis suggested that there would be no significant differences between the empirically observed and permuted temporal generalization matrices. To test this hypothesis, we generated a null distribution map by utilizing a permutation-based technique. Specifically, this involved randomly reallocating participants among various groups and then computing the mean values of these groups and their corresponding differences. This permutation procedure was iterated 1,000 times, resulting in a bimodal distribution of maximal and minimal values. The statistical significance was then determined based on a pre-established criterion: values above 0.005 below 0.995 were deemed to be statistically non-significant, while those surpassing this threshold were classified as significant. Finally, to quantify the statistical differences in temporal points relevant to decoding capabilities among the proposed methods applied to multi-class classification, paired t-tests with FDR correction were applied to the distributions of decoding performance collected from the participants.

## Results

### One-vs-rest classification and fusion analysis

**Fig. 4:**
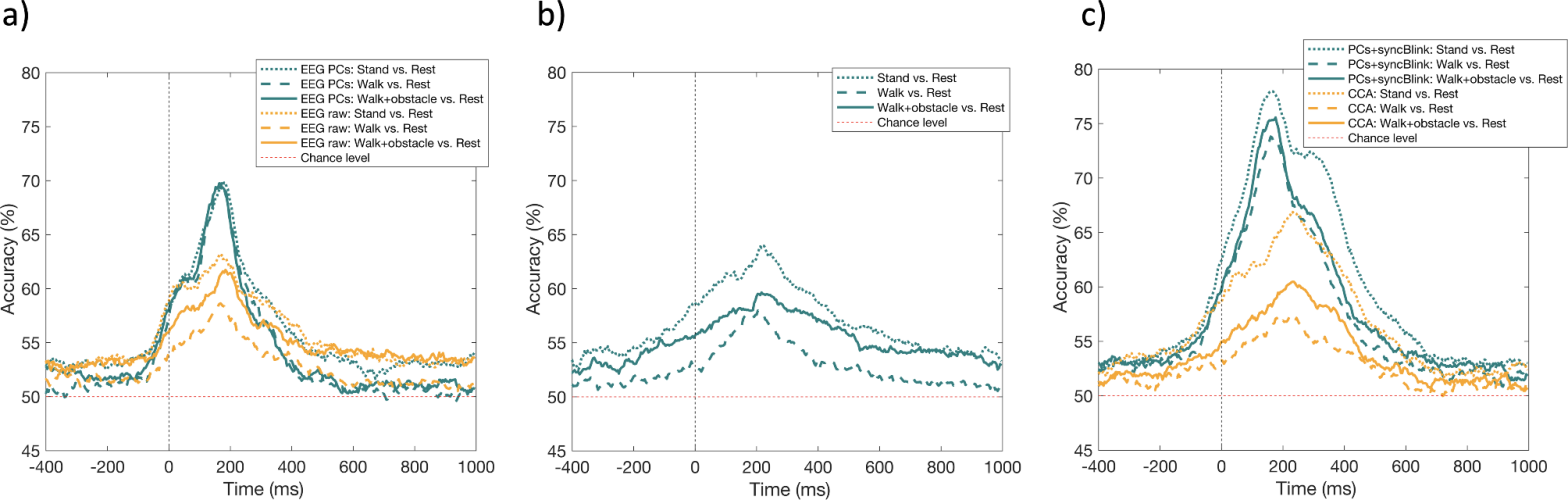
Comparisons of decoding accuracy for different data sources and fusion methods. (**a**) EEG data is represented through principal components and in raw form. (**b**) Synchronized eye-blink data. Fusion results are presented in (**c**). Fusion results showcasing a combination of principal components with synchronized blink data; and CCA variables derived from both raw EEG and synchronized blink data. Reference indicators: The dashed red line denotes chance levels. The densely dashed lines illustrate the stand vs. rest classification, the loosely dashed lines indicate the walk vs. rest classification, and the solid lines demonstrate the walk with obstacles vs. rest classification.

The results depicted in Fig. 4 provide a detailed examination of the decoding accuracy derived from various data sources and multimodal integration strategies. In Fig. 4(a), we examine the decoding accuracy of raw EEG signals and their PCs across different experimental conditions. The PCs of the EEG data, referred to as " pcEEG”, consistently exhibit a more stable accuracy trend across all conditions, achieving an average decoding accuracy of 70%, with a pronounced peak in accuracy noted within the 164–176 ms temporal window. In comparison, the raw EEG data display a more variable accuracy trend, with the highest accuracy of 63% observed in the ‘stand vs. rest’ condition. This is followed by 63% accuracy for the ‘walk with obstacles vs. rest’ condition, and 59% for the ‘walk vs. rest’ condition. These accuracies are all observed within a temporal window of 164–184 ms.

Figure 4(b) details the decoding accuracy for synchronized eye-blink data, referred to as " syncBlink”, showing a pattern akin to that observed in raw EEG data, with a peak accuracy around the time window of 204-224ms. The highest decoding accuracy observed is 64% in the ‘stand vs. rest’ condition, followed by 60% for ‘walk with obstacles vs. rest’, and finally 58% for ‘walk vs. rest’. However, it is important to note that synchronized eye-blink data shows a lower decoding accuracy compared to pcEEG. In Fig. 4(c), we examine the impact of various fusion methods on decoding accuracy. The findings reveal that fusion strategies significantly surpass single-modality techniques in terms of performance. Notably, when pcEEG is combined with syncBlink, an approach termed “pcEEG+,” there is a marked enhancement in accuracy. Within a temporal window of 160–176 ms, this integrated method achieves remarkable decoding accuracies: approximately 78% in the ‘stand vs. rest’ scenario, 76% in ‘walk with obstacles vs. rest’, and 74% in ‘walk vs. rest’. Conversely, the CCA method demonstrates lower accuracy across all conditions compared to pcEEG+. Specifically, CCA attains an accuracy of 67% for ‘stand vs. rest’, 61% for ‘walk with obstacles vs. rest’, and 57% for ‘walk vs. rest’, all within a temporal window of 224–236 ms. Based on these findings, pcEEG, syncBlink, and pcEEG+ syncBlink were selected for further investigation due to their superior decoding accuracy across various conditions.

**Fig. 5:**
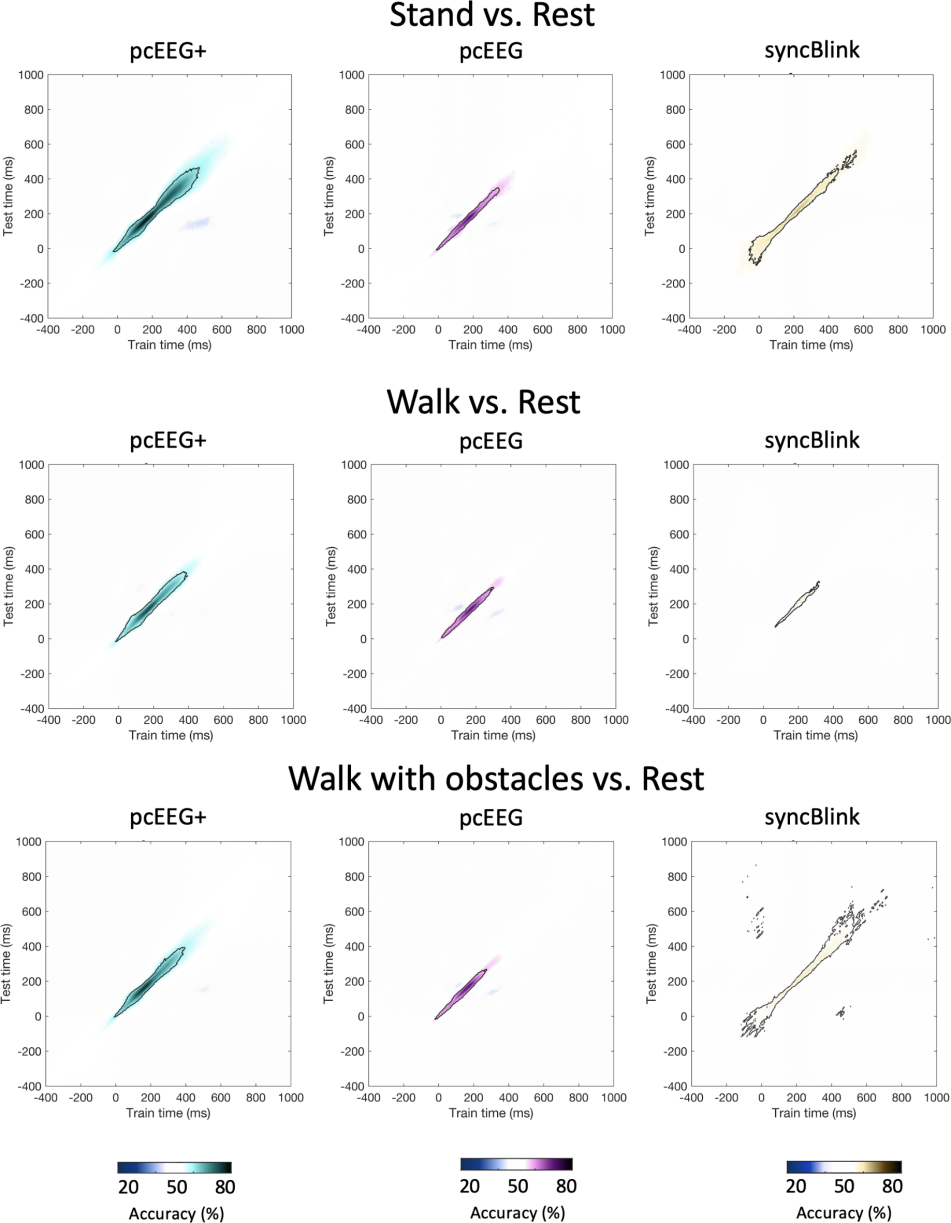
Illustration of temporal decoding for outdoor locomotor tasks categorized into stand, walk, and walk with obstacles conditions, each trained to differentiate from the rest (i.e., stand vs. rest, walk vs. rest, and walk with obstacles vs. rest) using data pcEEG+, pcEEG, and syncBlink. The color gradient represents decoding scores, with white signifying chance level. Significant clusters, as identified through the Pixel-based permutation test, are highlighted by black contour lines.

In Fig. 5, the temporal generalization matrix illustrates the decoding accuracy in a time-by-time format, from − 400 ms before to 1000 ms after the blink peak. This is an extension of the classical decoding analysis, where a classifier is not just trained and tested at the same time point, but its generalization capability across various time points is evaluated. The narrow main diagonal pattern in the matrix signals a temporally localized decoding accuracy, which can be construed as a consistent neural processing pattern for these locomotor conditions. This temporal consistency was observed across all methods— pcEEG+, pcEEG, and syncBlink— starting at the blink peak (0 ms) and continuing for at least 350 ms. Significantly, pcEEG+ exhibited superior performance, evidenced by its more intense color in the temporal generalization matrix across all conditions. Both pcEEG and syncBlink methods also displayed significant diagonal clusters, as marked by the black contour lines, though these were less pronounced compared to pcEEG+.

**Fig. 6:**
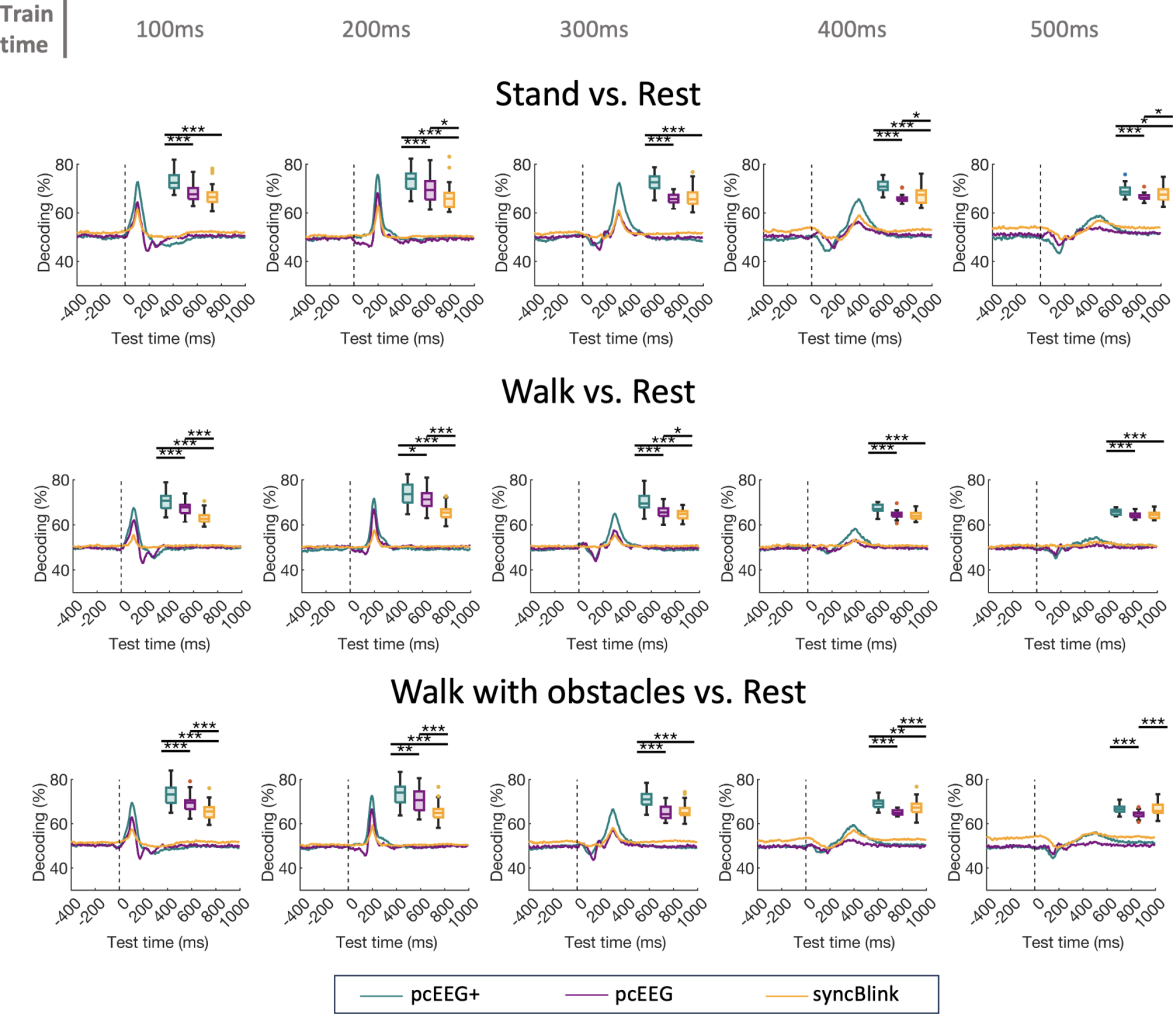
Temporal decoding accuracy scores derived from the temporal generalization matrix, trained at 100, 200, 300, 400, and 500 ms. A comparative analysis of these decoding accuracy scores across each method is undertaken, with statistical significance determined through a t-test. Significance levels are denoted as * for *p* < 0.05, ** for *p* < 0.01, and *** for *p* < 0.001.

To provide additional context to these findings, Fig. 6 illustrates the effectiveness of three methods—pcEEG, syncBlink, and pcEEG+—across three distinct locomotor conditions: standing, walking, and walking with obstacles. The binary classification performance of these methods was assessed at various training time points (100, 200, 300, 400, and 500 ms). Using a paired t-test with FDR correction for statistical validation, our results indicate a consistent superiority of the pcEEG+ method in distinguishing between each condition versus rest. Specifically, in the stand vs. rest condition, pcEEG + demonstrated significantly higher efficacy compared to both pcEEG and syncBlink across all time points (*p* < 0.001 at most time points). This trend was similarly observed in the walk vs. rest and walk with obstacles vs. rest conditions, with pcEEG+ consistently surpassing the other two methods. While the differences between pcEEG and syncBlink were not always statistically significant, they became pronounced at specific time points in each condition (e.g., *p* < 0.05 at 400 ms and 500 ms in stand vs. rest). These findings suggest that integrating principal components of EEG with the synchronization of eye blinks improves the decoding of neurophysiological states in response to various physical activities. The consistent performance of pcEEG+ across various conditions and time points highlights its potential as a robust tool for EEG data analysis.

### Multi-class classification: assessing generalizability

**Fig. 7:**
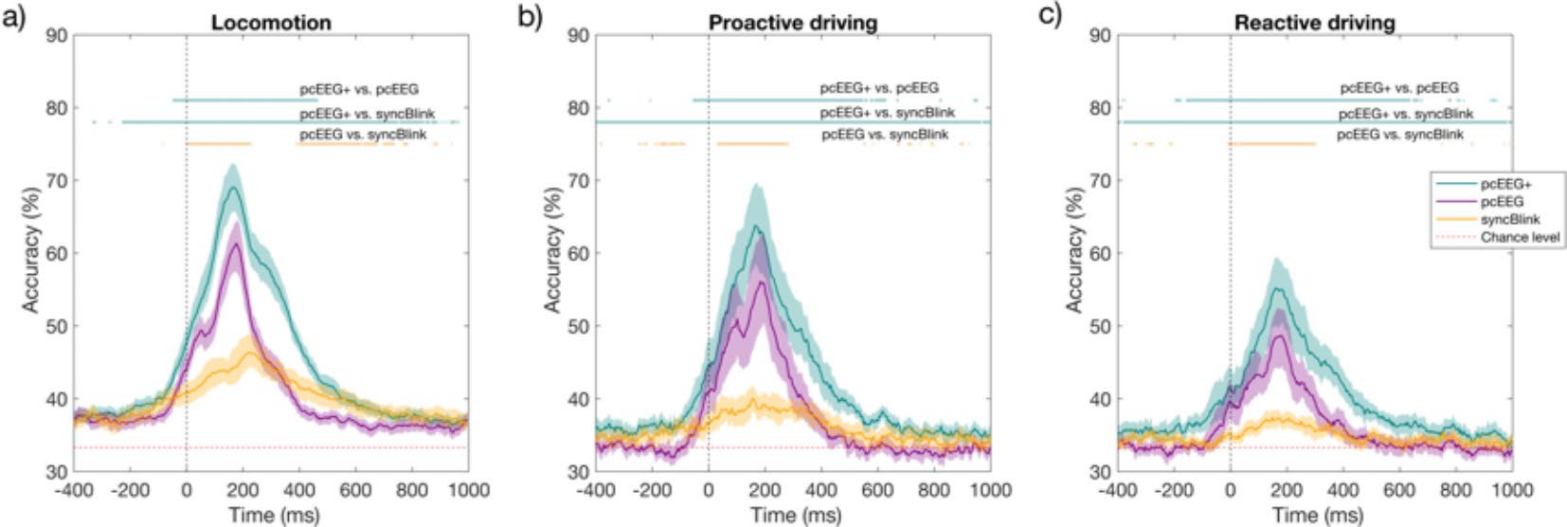
Multi-class decoding/classification accuracy across pcEEG+, pcEEG, and syncBlink: (**a**) locomotor task dataset, (**b**) proactive driving dataset and (**c**) reactive driving dataset. The dashed red line at the bottom represents the chance-level accuracy. The shaded area denotes the bootstrapped 95% confidence interval. Statistically significant differences (*p* < 0.05, FDR-corrected) between methods are denoted by the upper colored lines.

In this section, we evaluate the effectiveness of our algorithms in a multi-classification task encompassing three distinct locomotor activities: standing, walking, and walking with obstacles. Additionally, we utilize replication datasets from both proactive and reactive driving scenarios, categorized into three levels of difficulty: low, middle and high. Figure 7; Table 1 present a comparative analysis of multi-class decoding accuracy, sensitivity, and specificity scores across three distinct datasets: locomotor tasks, proactive driving and reactive driving. These datasets were analyzed using three analytical methods: pcEEG+, pcEEG and syncBlink. In each dataset, the decoding accuracy for all three methods demonstrably surpassed the chance level, albeit with varying peaks of accuracy. Specifically, within the locomotor task dataset (as depicted in Fig. 7a), the pcEEG+ method yielded notably high decoding accuracy, sensitivity, and specificity scores of 69.0%, 69.0%, and 84.5%, respectively, observed at 168 ms. This performance numerically surpasses that obtained with the pcEEG alone (accuracy = 61.4%, sensitivity = 61.4%, specificity = 80.7%) at 176 ms and the syncBlink alone (accuracy = 46.3%, sensitivity = 46.3%, specificity = 73.2%), which occurred at 220 ms. These observed disparities in accuracy were statistically confirmed through a paired t-test (*p* < 0.05, FDR-corrected).

To validate the adaptability of the pcEEG+ method in different motor-cognitive domains, the robustness of the method was confirmed across varied driving tasks. For instance, within the proactive and reactive driving datasets (refer to Fig. 7b and c), the pcEEG+ approach maintained superior performance over other methods. It achieved mean accuracy, sensitivity, and specificity values of 63.8%, 63.8%, and 81.9%, respectively, at 164 ms for the proactive dataset, and 55.2%, 55.2%, and 77.6% at 160 ms for the reactive dataset. When utilizing the pcEEG approach, the classification metrics were 56.1% for accuracy, 56.1% for sensitivity and 78.0% for specificity at 184 ms in the proactive driving dataset, and 48.7% for accuracy, 48.7% for sensitivity, and 74.3% for specificity at 176 ms in the reactive driving dataset. The syncBlink method registered values of 41.1% for accuracy, 41.1% for sensitivity, and 70.0% for specificity at 156 ms in the proactive driving dataset, and values of 37.4% for accuracy, 37.4% for sensitivity, and 68.7% for specificity at 148 ms in the reactive driving dataset. These outcomes were also verified to be statistically significant (*p* < 0.05, FDR-corrected).

**Table 1:** Comparison of classification performance across multiple computational techniques.

Dataset	Method	Apex time	Acc(%)	Sen(%)	Spec(%)
Locomotion	*pcEEG+*	168	69.0	69.0	84.5
	*pcEEG*	176	61.4	61.4	80.7
	*syncBlink*	220	46.3	46.3	73.2
Proactive	*pcEEG+*	164	63.8	63.8	81.9
	*pcEEG*	184	56.1	56.1	78.0
	*syncBlink*	156	40.1	40.1	70.0
Reactive	*pcEEG+*	160	55.2	55.2	77.6
	*pcEEG*	176	48.7	48.7	74.3
	*syncBlink*	148	37.4	37.4	68.7

In sum, the pcEEG+ strategy demonstrated an accuracy improvement of 7.6% and 22.7% over the pcEEG-only and syncBlink-only methods, respectively, in the locomotor task data. Similarly, the proactive driving data showed improvements of 7.7% and 23.7% over these methods; for the reactive driving data, improvements were 6.5% and 17.8%. Additionally, the pcEEG-only outperformed the syncBlink-only approach, achieving improvements of 15.1%, 16.0%, and 11.3% in the locomotor task, proactive, and reactive driving datasets, respectively. The fusion approach effectively leverages the complementary characteristics of EEG and eye-blink data to enhance decoding accuracy.

**Fig. 8:**
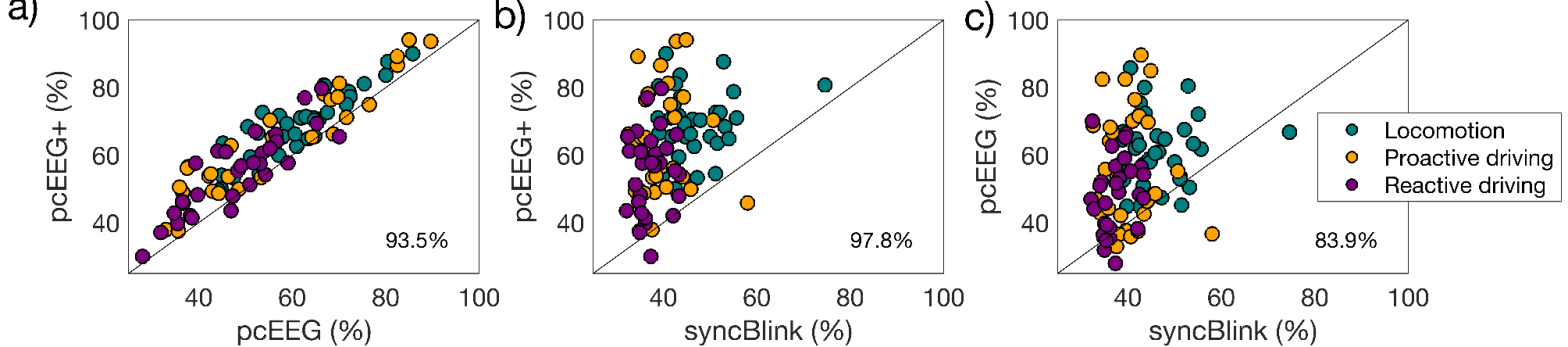
Comparative scatter plots showing classification accuracies of various methods: (**a**) pcEEG+ vs. pcEEG, (**b**) pcEEG+ vs. syncBlink, (**c**) pcEEG vs. syncBlink. The black diagonal line represents an equal accuracy between the two methods. Circles positioned above the diagonal line indicate an improvement in classification accuracy. The circles are color-coded to represent distinct datasets: teal for locomotor tasks, orange for proactive driving, and purple for reactive driving. The percentage values indicate the proportion of participants demonstrating comparable or improved results.

Figure 8 offers an exhaustive comparative analysis of the classification accuracies attained via multiple methods: pcEEG+, pcEEG, and syncBlink. The results employ scatter plots to graphically represent these accuracies, with the black diagonal line serving as a reference for equal performance between the methods under comparison. In Figs. 8(a) and 7(b), most of the data points—represented by teal, orange, and purple circles corresponding to locomotor tasks, proactive and reactive driving, respectively—are situated above this diagonal line. This spatial arrangement serves as empirical evidence that the multimodal fusion approach (pcEEG+) outperforms the unimodal methods, such as pcEEG and syncBlink, in terms of classification accuracy. A remarkable 97.8% and 93.5% of the participants demonstrated improved classification performance when leveraging the fusion approach (pcEEG+) compared to syncBlink and pcEEG, respectively. Figure 8(c) shows that pcEEG could achieve superior results compared to using the syncBlink approach; it was observed that 83.9% of participants presented improved classification.

## Discussion

In this study, we introduced a new method focusing on the synchronization-based fusion of EEG and eye-blink data. This multimodal approach presents a significant advance over single-modality techniques, which are typically limited in decoding performance during dynamic tasks such as locomotion^43^. The contribution of this study expands the existing body of knowledge by demonstrating how integrating pcEEG with syncBlink significantly enhances decoding accuracy across various motor tasks over single-modality methods. Previous analyses^16^ with these datasets demonstrated that eye-blink-related EEG activity, particularly in the alpha and theta bands, reflects the cognitive demands of locomotion. We found that alpha and theta band activities, associated with eye blinks, were modulated by task complexity, indicating an intricate relationship between neural activity and ocular events. Building on these findings, the new contribution of this research lies in the multimodal integration approach, where we demonstrate that combining EEG principal components with synchronized eye-blink data significantly enhances decoding accuracy. This multimodal fusion represents a marked improvement over the existing single-modality approaches.

The findings herein provide robust evidence that the principal components from EEG data (pcEEG) consistently exhibit more stable accuracy trends across all conditions, with an average decoding accuracy of 70%, significantly higher than raw EEG data. This improvement highlights the importance of PCA in reducing the dimensionality of high-variance EEG datasets while preserving essential information^44^. The PCA effectively removes noise and redundancy, which is critical given the complexity of EEG signals in locomotor tasks, and improves overall model performance by 8.33% compared to raw data. In contrast, the standalone use of synchronized eye-blink data (syncBlink) results in lower decoding accuracy compared to pcEEG. This outcome underscores the significance of temporal synchronization in multimodal data integration, as timing discrepancies between EEG and eye activity can affect decoding effectiveness.

The integration of pcEEG and syncBlink (pcEEG+), which forms the crux of the study, surpasses the accuracy of single-modality techniques. The pcEEG+ approach demonstrates a remarkable decoding accuracy of about 74–78% across different scenarios, reinforcing the idea that multimodal fusion can lead to a more nuanced and comprehensive understanding of cognitive states. This concept aligns with existing literature, where the integration of EEG and eye-tracking has been shown to enhance classification accuracy across various conditions^27,28,43,45–47^. Vortmann et al.^46^ and Cheng et al.^47^, for example, demonstrated the effectiveness of combining EEG and eye-tracking for better attention classification and motion imagery in BCIs, highlighting the practical benefits of this multimodal approach. While extant literature predominantly concentrates on classification methodologies, our investigation uncovers the potential of decoding strategies to unravel the complex interplay between EEG patterns and eye blinks within cognitive processes. It suggests the complementary nature of EEG and eye-blink data in cognitive state decoding, aligning with the broader literature on the enhanced efficacy of multimodal data fusion in neuroscience, a concept supported by^19,20^. The superior accuracy manifested by the pcEEG+ method, in comparison to the individual modalities, is congruent with the established notion of multimodal data fusion in neuroscience, offering a more comprehensive and detailed perspective of brain activity.

Furthermore, our study emphasizes the temporal specificity of accuracy peaks within certain post-blink windows, highlighting the criticality of precise temporal alignment in multimodal analyses. The temporal discrepancy between peak EEG activities and eye blinks emerges as a pivotal factor in achieving optimal decoding accuracy. This concept is buttressed by literature emphasizing the synchronization of EEG and eye-tracking data^48^. The principle of temporal synchronization, as evidenced by the optimal decoding windows identified in our study, resonates with the findings of Dimigen et al.^49^. Their work on regression-based analysis of combined EEG and eye-tracking data lays a theoretical foundation for elucidating the interaction between these modalities, particularly in terms of their temporal dynamics.

Additionally, the temporal generalization dynamics underlying locomotor tasks are further elucidated in this study. The temporal generalization matrix, a key result from our study, offers a novel perspective on how the brain’s neural processes vary with time in response to different cognitive states and locomotor tasks. This matrix reveals a temporally localized decoding accuracy, particularly pronounced in the multimodal fusion approach (pcEEG+), starting at the blink peak and continuing for at least 350 ms. This finding is crucial, as it not only affirms the consistency of neural processing patterns across different tasks but also underlines the temporal dynamics of neural activity. In the context of existing literature, the concept of a temporal generalization matrix aligns with the understanding that neural responses to stimuli are not static but evolve over time. Studies have shown that temporal patterns in EEG data can provide significant insights into brain activity and cognitive processes^3,20^. The temporal generalization matrix extends this understanding by demonstrating how the decoding accuracy of integrated EEG and eye-blink data varies over time, highlighting the dynamic nature of neural processing. The superior performance of pcEEG+ over a single modality in the temporal generalization matrix can be attributed to the enhanced signal-to-noise ratio and the richer information content achieved through the integration of EEG and eye-blink data. This aligns with previously mentioned studies that highlighted the importance of combining EEG and eye data for cognitive state classification^27,28,43,45–47^.

The study further amplifies its analytical rigor by evaluating the generalizability of our algorithm through a multi-classification task that encompasses three distinct datasets: locomotion datasets and two datasets from driving simulator studies (proactive and reactive driving). The fusion approach (pcEEG+) consistently surpasses the individual modality in all datasets, thereby attesting to its robustness and generalizability. The improvements of 7.6% and 22.7% over pcEEG-only and syncBlink-only in locomotor tasks underscore the effectiveness of this fusion approach. These improvements are also pronounced in proactive and reactive driving scenarios, demonstrating the robustness of this approach. The enhancement in accuracy can be recognized by the method’s ability to harness the complementary characteristics of EEG and eye-blink data, effectively capturing a more comprehensive picture of neural-ocular activity related to locomotor tasks and driving scenarios. The scatter plots in Fig. 8 demonstrate the superiority of the fusion approach over unimodal methodologies, aligning with the literature on multimodal data fusion benefits^27,28,43,45–47,50,51^. The distinct performance patterns observed in proactive and reactive driving tasks further emphasize the adaptability and robustness of the pcEEG+ method in varying experimental settings.

While the study provides notable advancements in the field of computational neuroscience, particularly in decoding using combined EEG and eye blink data, there are inherent limitations that warrant acknowledgment. For example, the selected tasks, such as outdoor walking and driving tasks using controlled simulations, may not reflect the complexities encountered in real-world settings. These tasks may not encompass the full range of challenges posed by natural environments. More complex real-world tasks or multitasking scenarios may introduce greater variability in brain activity and blink behavior, which could complicate the synchronization of EEG and eye blink signals. This highlights the need for further exploration into how this method can be applied to more naturalistic and multitasking environments. Future research should, therefore, expand the scope to include a broader range of cognitive states and external stimuli. Additionally, exploring the integration of other modalities, such as functional Magnetic Resonance Imaging (fMRI), Electromyography (EMG), or Magnetoencephalography (MEG), could provide a more comprehensive view of brain activity. Lastly, while the study effectively demonstrates the robustness of its fusion algorithms in the domains of locomotor tasks and proactive driving, the generalizability of these algorithms to additional cognitive landscapes remains empirically unverified. Consequently, forthcoming research endeavors should prioritize the application of these fusion algorithms within diverse cognitive contexts, thereby further substantiating their universal applicability and robustness. We also recognize the importance of exploring other classification methods and should consider their implementation in future studies.

## Conclusion

The present study constitutes a substantial contribution to computational neuroscience by significantly improving the accuracy of cognitive state decoding in dynamic tasks through the fusion of EEG and synchronized eye-blink data from EEG (pcEEG+). This integration leads to a notable increase in decoding accuracy, consistently surpassing single-modality techniques across various scenarios and datasets, thereby highlighting the advantages of multimodal data fusion. Moreover, our study highlights the critical importance of temporal synchronization, with the temporal generalization matrix revealing a distinct decoding accuracy pattern, especially post-blink, for at least 350 ms. This underlines the dynamic nature of neural processing. Furthermore, the generalizability of our method was affirmed across various tasks, including locomotor, proactive driving, and reactive driving scenarios, where pcEEG+ consistently outperformed unimodal methods. This suggests that the integration of EEG and eye-blink data is not only effective in decoding cognitive states but also robust across different experimental contexts, offering a promising avenue for advancing our understanding of brain activity dynamics.

## Data Availability

The datasets generated during the current study are available from the corresponding author on reasonable request.
